# Supplementary material for: The constant philopater hypothesis: a new life history invariant for dispersal evolution
Source: J Evol Biol. 2015 Oct 31;29(1):153–66. doi: 10.1111/jeb.12771 (PMC4738439; doi:10.1111/jeb.12771)
Supplement: Supplementary file 1 — Appendix S1 Ecological dynamics. Appendix S2 Reproductive success. Appendix S3 Stable class frequencies and reproductive values. Appendix S4 Fitness. Appendix S5 Selection gradient. Appendix S6 Relatedness. Appendix S7 Convergence stability. Appendix S8 Tables S1 & S2. Table S1 Recursion equations and coefficients of consanguinity under maternal control. Table S2 Coefficient of consanguinity under offspring control. [file JEB-29-153-s001.pdf]

Supporting information to: "The constant philopater hypothesis: a new life history invariant for dispersal evolution"

Antonio M. M. Rodrigues[1,2,*], Andy Gardner[3]

1. Department of Zoology, University of Cambridge, Downing Street, Cambridge CB2 3EJ, United Kingdom.

2. Wolfson College, Barton Road, Cambridge CB3 9BB, United Kingdom.

3. School of Biology, University of St Andrews, St Andrews KY16 9TH, United Kingdom.

[*] Corresponding author, email: ammr3@cam.ac.uk

Contents:
Appendix S1. Ecological dynamics
Appendix S2. Reproductive success
Appendix S3. Stable class frequencies and reproductive values
Appendix S4. Fitness
Appendix S5. Selection gradient
Appendix S6. Relatedness
Appendix S7. Convergence stability
Appendix S8. Table S1 & S2
References

Appendix S1. Ecological dynamics

Here, we follow the ecological dynamic of patches resource-availability outlined in the main text. This can be described by a transition matrix, which is given by

$$\mathbf{P} = \begin{pmatrix} \eta_{11} & \cdots & \eta_{n_\mathrm{p}1} \\ \cdots & \ddots & \cdots \\ \eta_{1n_\mathrm{p}} & \cdots & \eta_{n_\mathrm{p}n_\mathrm{p}} \end{pmatrix}, \qquad \text{(S1.1)}$$

where η_{ij} is the probability that a type-i patch becomes a type-j patch the next season. At ecological equilibrium, there is a stable frequency of the different types of patches in the population, which is given by the elements of the right-eigenvector of matrix **P**. We denote the frequency of type-i patches at equilibrium by p_i. If we define a random variable T_t, denoting the state of a focal patch in season t, then the coefficient of correlation between two successive seasons, denoted by τ, is defined as $\tau \equiv \mathrm{cov}(T_t,T_{t+1})\sqrt{\ }(\mathrm{var}(T_t)\mathrm{var}(T_{t+1}))$, where $-1 \leq \tau \leq 1$ (Rodrigues and Gardner 2012). An environment is temporally stable when $\tau = 1$, temporally unpredictable when $\tau = 0$, and locally seasonal when $\tau = -1$.

Appendix S2. Reproductive success

The probability that a focal juvenile wins a breeding site is $k_{ta}(x,z) = 1/(\sum_{b\in I}F_{bt}\sigma_{bta}(1-x_{bta})+\sum_{q\in T}p_q(\sum_{b\in I}F_{bq}\sigma_{bqa}z_{bqa})(1-c))$, with $a \in\{f,m\}$, $I = \{1, 2, \ldots, n\}$, $T = \{1, 2, \ldots, n_p\}$, $\sigma_{btf} = 1-\sigma_{bt}$, and $\sigma_{btm} = \sigma_{bt}$; and where: F is the fecundity of a focal mother; x the probability of dispersal of a focal offspring; and z the population average probability of dispersal of an offspring. The reproductive success of a rank-i mother in a type-t patch through her successful daughters that become rank-j mothers in a type-q patch is $w_{itf\to jqf} = F_{it}(1-\sigma_{it})((1-x_{itf})k_{tf}(x,z)\eta_{tq}+x_{itf}(1-c)\sum_{e\in T}p_ek_{ef}(x,z)\eta_{eq})(1-\phi)$, where ϕ is the fraction of genes a daughter inherits from her father. The reproductive success of a rank-i mother in a type-t patch through her successful sons that mate with rank-j mothers in type-q patches is $w_{itf\to jqm} = F_{it}(1-\sigma_{it})((1-x_{itf})k_{tf}(x,z)\eta_{tq}+x_{itf}(1-c)$ $\sum_{e\in T}p_ek_{qf}(x,z)\eta_{eq})\mu$, where μ is the fraction of genes a son receives from his mother. The reproductive success of a rank-i father in a type-t patch (i.e. a father that mates with a rank-i mother in a type-t patch) through his successful daughters that become rank-j mothers in type-q patches is $w_{itm\to jqf} = F_{it}\sigma_{it}((1-x_{itm})k_{tm}(x,z)\eta_{tq}+x_{itm}(1-c)\sum_{e\in T}p_ek_{em}(x,z)\eta_{eq})\phi$. The reproductive success of a rank-i father in a type-t patch through his successful sons that mate with rank-j mothers in type-q patches is $w_{itm\to jqm} = F_{it}\sigma_{it}((1-x_{itm})k_{tm}(x,z)\eta_{tq} +x_{itm}(1-c))\sum_{e\in T}p_ek_{em}(x,z)\eta_{eq})(1-\mu)$. In the asexual reproduction model, there is no male component in the reproductive success expressions, in which case we drop the subscript 'f' from the reproductive success expressions of females, and we set $\phi = 0$.

Appendix S3. Stable class frequencies and reproductive values

The expressions of the reproductive success of individuals define a transition matrix, which is given by

$$\mathbf{A} = \begin{pmatrix} (w_{itf\to jqf})_{n.n_p\times n.n_p} & (w_{itm\to jqf})_{n.n_p\times n.n_p} \\ (w_{itf\to jqm})_{n.n_p\times n.n_p} & (w_{itm\to jqm})_{n.n_p\times n.n_p} \end{pmatrix}. \qquad \text{(S3.1)}$$

The elements of the right-eigenvector of matrix **A** (corresponding to the leading eigenvalue) give the frequency of each class (Taylor & Frank 1996; Grafen 2006), which is $u_{it} = 1/(n.n_p)$, for all $i \in I$, $t \in T$. The elements of the left-eigenvector of matrix **A** (corresponding to the leading eigenvalue) give the reproductive values for individuals of each class (Fisher 1930; Taylor & Frank 1996; Grafen 2006). The class-reproductive values are $c_f = \phi/(\phi+\mu)$ and $c_m = \mu/(\phi+\mu)$. Under asexual-reproduction, $c_f = 1$.

Appendix S4. Fitness

The fitness of a focal individual is the sum of its different reproductive success components weighted by corresponding reproductive values, all divided by the mean reproductive value of the focal class. This is $W_{itf} = (\sum_{j \in I}\sum_{q \in T} w_{itf \to jqf} v_{jqf} + \sum_{j \in I}\sum_{q \in T} w_{itf \to jqm} v_{jqm})/v_{itf}$, and $W_{itm} = (\sum_{j \in I}\sum_{q \in T} w_{itm \to jgf} v_{jgf} + \sum_{j \in I}\sum_{q \in T} w_{itm \to jqm} v_{jqm})/v_{itm}$, for females and for males, respectively. The fitness of a random individual is given by the sum of fitness weighted by the frequency and reproductive value of each class. This is $w = \sum_{j \in I}\sum_{q \in T} u_{iqf} v_{iqf} W_{iqf} + \sum_{j \in I}\sum_{q \in T} u_{iqm} v_{iqm} W_{iqm}$.

Appendix S5. Selection gradient

The selection gradient is given by the slope of fitness on the breeding value of the focal recipient (i.e. the beneficiary of social behaviours): $dw/dg_{it} = \sum_{j \in I}\sum_{q \in T} u_{jqf} v_{iqf} (\partial W_{jqf}/\partial x_{it})(dG_{jqf}/dg_{it}) + \sum_{j \in I}\sum_{q \in T} u_{jqm} v_{jqm} (\partial W_{jqm}/\partial x_{it})(dG_{jqm}/dg_{it})$, where: the slope of fitness on phenotypes give the marginal fitness effect of the behaviour; and the slope of the actor's (i.e. the enactor of a social behaviour) breeding value (i.e. the heritable component of an actor's phenotype), denoted by G, on the focal recipient's breeding value (i.e. the heritable component of a focal recipient's phenotype), denoted by g, gives the kin selection relatedness coefficients (Taylor & Frank 1996; Frank 1998; Rodrigues and Gardner 2013). Expanding the RHS of this equation, we find that the condition for the evolution of a slightly higher dispersal rate of a daughter is $-r_{itf}\omega_t\upsilon_t + (1-c) r_{itf} \sum_{q \in T} p_q \omega_q \upsilon_q + \omega_t \upsilon_t h_f \sum_{j \in I} U_{itf} \rho_{ijtf} > 0$, whereas the condition for the evolution of a slightly higher dispersal rate of a son is $-r_{itm}\omega_t\upsilon_t + (1-c) r_{itm} \sum_{q \in T} p_q \omega_q \upsilon_q + \omega_t \upsilon_t h_f \sum_{j \in I} U_{itm} \rho_{ijtm} > 0$, where: $\omega_{tf} = k_{tf}(z,z)$ is the probability a single female wins a breeding site in a type-t patch; $\omega_{tm} = k_{tm}(z,z)$ is the probability that a single male wins a breeding site in a focal type-t patch; $h_{tf} = k_{tf}(z,z)\sum_{i \in I} F_{it}(1-\sigma_{it})(1-z_{itf})$ is the probability a random juvenile female after dispersal is born in the focal type-t patch; $h_{tm} = k_{tm}(z,z)\sum_{i \in I} F_{it}\sigma_{it}(1-z_{itm})$ is the probability that a random juvenile male after dispersal is born in the focal type-t patch; $U_{jqf} = (F_{jq}(1-\sigma_{jq})(1-z_{jqf}))/(\sum_{b \in I} F_{bq}(1-\sigma_{bq})(1-z_{bqf}))$ is the frequency of the rank-j mother's daughters among the native daughters of a type-q patch; and $U_{jqm} = (F_{jq}\sigma_{jq}(1-z_{jqm}))/(\sum_{b \in I} F_{bq}\sigma_{bq}(1-z_{bqm}))$ is the frequency of the rank-j mother's sons among the native sons of a type-q patch. Under allomaternal control, we need to consider coefficients of relatedness between the allomother i and the offspring ϑ who are under the control of the allomother, in which case $r_{it} = r_{i\vartheta t}$. To determine the selection gradient for the evolution of the sex allocation strategy, we follow the methodology outlined for the evolution of dispersal. However, we assume that the sex allocation strategy σ is an evolving trait rather than a parameter. Hamilton's rule for the evolution of the sex allocation strategy is given by equation (4) and (5).

Appendix S6. Relatedness

We assume a neutral population, and for each of the reproductive systems we define recursion equations for the coefficients of consanguinity in successive generation between juveniles, which we then solve for equilibrium. The coefficients of consanguinity allow us to derive the coefficients of relatedness between interacting individuals (Bulmer 1994, Rodrigues and Gardner 2013). We focus on three coefficients of consanguinity: (1) the coefficient of consanguinity between

opposite-sex offspring (denoted by f); (2) the coefficient of consanguinity between female offspring (denoted by γ); and (3) the coefficient of consanguinity between male offspring (denoted by η). All the recursion equations have the form $X_t' = \sum_{q\in T}\pi(q|t)(P_{SqX}Y_q + (1-P_{SqX})Z_q)$, where: $\pi(q|t)$ is the probability that a type-t patch was a type-q patch in the previous generation; X is a coefficient of consanguinity (i.e. f, γ, or η); $P_{Sqf} = \sum_{i\in I}((F_{iq}(1-\sigma_{iq})/\sum_{j\in I}F_{jq}(1-\sigma_{jq}))(F_{iq}\sigma_{iq}/\sum_{j\in I}F_{jq}\sigma_{jq}))$, is the probability that two opposite-sex offspring sampled at random before dispersal are siblings; $P_{Sq\gamma} = \sum_{i\in I}(F_{iq}(1-\sigma_{iq})/\sum_{j\in I}F_{jq}(1-\sigma_{jq}))^2$ is the probability that two female offspring sampled at random before dispersal are siblings; $P_{Sq\eta} = \sum_{i\in I}(F_{iq}\sigma_{iq}/\sum_{j\in I}F_{jq}\rho_{jq})^2$ is the probability that two male offspring sampled at random before dispersal are siblings; Y_q is the probability that two siblings share genes in common; and Z_q is the probability that two non-siblings share genes in common. The variables X, Y, and Z, depend on the type of reproduction, on the type of inheritance, and on the type of patch. In table 1 and 2 we define these variables for each case. The coefficients of consanguinity can then be used to define the coefficients of relatedness between interacting individuals. The relatedness between: (1) a mother and her daughters is $r_{MD} = p_{MD} / p_M$; (2) a mother and her sons is $r_{MS} = p_{MS} / p_M$; (3) a mother and a daughter the other mother is $r_{MF} = p_{MF} / p_M$; (4) a mother and a son of another mother is $r_{MM} = p_{MM} / p_M$.

Appendix S7. Convergence stability

To determine if a pair of optimal dispersal strategies is convergence stable (CS; Christiansen 1991; Eshel 1996; Taylor 1996) we define the matrix:

$$\left.\begin{pmatrix} \dfrac{\partial}{\partial z_{\mathrm{nn_p}}}\left(\left.\dfrac{\partial W}{\partial g_{\mathrm{nn_p}}}\right|_{x_{\mathrm{nn_p}}=z_{\mathrm{nn_p}}}\right) & \vdots & \dfrac{\partial}{\partial z_{11}}\left(\left.\dfrac{\partial W}{\partial g_{\mathrm{nn_p}}}\right|_{x_{\mathrm{nn_p}}=z_{\mathrm{nn_p}}}\right) \\ \vdots & \ddots & \vdots \\ \dfrac{\partial}{\partial z_{\mathrm{nn_p}}}\left(\left.\dfrac{\partial W}{\partial g_{11}}\right|_{x_{11}=z_{11}}\right) & \vdots & \dfrac{\partial}{\partial z_{11}}\left(\left.\dfrac{\partial W}{\partial g_{11}}\right|_{x_{11}=z_{11}}\right) \end{pmatrix}\right|_{z_{11}=z_{11}^*,\ldots,z_{\mathrm{nn_p}}=z_{\mathrm{nn_p}}^*} . \tag{S7.1}$$

The set of optimal strategies $(z_{11}^*,\ldots, z_{nn_p}^*)$ are convergence stable if the eigenvalues of matrix (F1) have negative real parts (Otto & Day 2007).

Appendix S8. Table S1 & S2

Table S1. Recursion equations and coefficients of consanguinity under maternal control

	$X' = \sum_{q \in T} \pi(q\|t)\ (P_{SqX}Y + (1-P_{SqX})Z)$			Coefficients of consanguinity				
	X	Y	Z	p_M	p_{MD}	p_{MF}	p_{MS}	p_{MM}
Asexual	γ	1	hhf	1	1	hhf	-	-
Sexual haploidy	f	$\frac{1}{2}+\frac{1}{2}\ h_f h_m f$	$\frac{1}{4}h_f h_f f+\frac{1}{2}h_f h_m f +\frac{1}{4}h_m h_m f$	1	$\frac{1}{2}p_M+\frac{1}{2}h_f h_m f$	$\frac{1}{2}h_f h_f f+ \frac{1}{2}h_f h_m f$	$\frac{1}{2}p_M+\frac{1}{2}h_f h_m f$	$\frac{1}{2}h_f h_f f+ \frac{1}{2}h_f h_m f$
Sexual diploidy	f	$\frac{1}{2}(\frac{1}{2}+\frac{1}{2}\ h_f h_m f) +\frac{1}{2}h_f h_m f$	$\frac{1}{4}h_f h_f f+\frac{1}{2}h_f h_m f +\frac{1}{4}h_m h_m f$	$\frac{1}{2}+\frac{1}{2}\ h_f h_m f$	$\frac{1}{2}p_M+\frac{1}{2}h_f h_m f$	$\frac{1}{2}h_f h_f f+ \frac{1}{2}h_f h_m f$	$\frac{1}{2}p_M+\frac{1}{2}h_f h_m f$	$\frac{1}{2}h_f h_f f+ \frac{1}{2}h_f h_m f$
Sexual haplodiploidy	f	$\frac{1}{2}(\frac{1}{2}+\frac{1}{2}\ h_f h_m f) +\frac{1}{2}h_f h_m f$	$\frac{1}{2}h_f h_f \gamma +\frac{1}{2}h_f h_m\, f$	$\frac{1}{2}+\frac{1}{2}\ h_f h_m f$	$\frac{1}{2}p_M+\frac{1}{2}h_f h_m f$	$\frac{1}{2}h_f h_f \gamma+ \frac{1}{2}h_f h_m f$	$\frac{1}{2}+\frac{1}{2}h_f h_m f$	$h_f h_f \gamma$
	γ	$\frac{1}{4}(\frac{1}{2}+\frac{1}{2}\ h_f h_m f)+ \frac{1}{2}h_f h_m f+\frac{1}{4}$	$\frac{1}{4}h_f h_f \gamma+\frac{1}{2}h_f h_m f +\frac{1}{4}h_m h_m \eta$					
	η	$\frac{1}{2}+\frac{1}{2}h_f h_m f$	$h_f h_f \gamma$					

Note: The coefficients of consanguinity are determined by solving the recursion equations for equilibrium: $\gamma' = \gamma$ under asexual reproduction; $f' = f$ under sexual reproduction with both haploid and diploid inheritance; and $\gamma' = \gamma$, $f' = f$, and $\eta' = \eta$, under sexual reproduction with haplodiploid inheritance. These coefficients are patch-type specific. We can then determine the coefficients of consanguinity between: a mother and herself (p_M); a mother and a daughter or a son (p_{MD} or p_{MS}); between a mother another mother's daughter or son (p_{MF} or p_{MM}).

Table S2. Coefficient of consanguinity under offspring control

	Coefficients of consanguinity					
	p_{OF}	p_{OFS}	p_{OFF}	p_{OM}	p_{OMS}	p_{OMM}
asexual	1	1	hhf	-	-	-
Sexual haploidy	1	$\frac{1}{2}p_{OF}+\frac{1}{2}h_f h_m f$	$\frac{1}{4}h_f h_f f+\frac{1}{2}h_f h_m f$ $+\frac{1}{4}h_m h_m f$	1	$\frac{1}{2} p_{OM}+\frac{1}{2}h_f h_m f$	$\frac{1}{4}h_f h_f f+\frac{1}{2}h_f h_m f$ $+\frac{1}{4}h_m h_m f$
Sexual diploidy	$\frac{1}{2}+\frac{1}{2}h_f h_m f$	$\frac{1}{2}(\frac{1}{2}+\frac{1}{2}h_f h_m f)$ $+\frac{1}{2}h_f h_m f$	$\frac{1}{4}h_f h_f f+\frac{1}{2}h_f h_m f$ $+\frac{1}{4}h_m h_m f$	$\frac{1}{2}+\frac{1}{2}h_f h_m f$	$\frac{1}{2}(\frac{1}{2}+\frac{1}{2}h_f h_m f)$ $+\frac{1}{2}h_f h_m f$	$\frac{1}{4}h_f h_f f+\frac{1}{2}h_f h_m f$ $+\frac{1}{4}h_m h_m f$
Sexual haplodiploidy	$\frac{1}{2}+\frac{1}{2}h_f h_m f$	$\frac{1}{4}(\frac{1}{2}+\frac{1}{2}h_f h_m f)$ $+\frac{1}{2}h_f h_m+\frac{1}{4}$	$\frac{1}{4}h_f h_f \gamma+\frac{1}{2}h_f h_m f$ $+\frac{1}{4}h_m h_m \eta$	1	$\frac{1}{2}+\frac{1}{2}h_f h_m f$	$h_f h_f \eta$

Note: Having derived the coefficients of consanguinity γ, f, and, η we can derive the coefficients of consanguinity between: a female offspring and herself (p_{OF}); a female offspring and her sisters (p_{OFS}); a female offspring and another mother's daughters (p_{OFF}); a male offspring and himself (p_{OM}); a male offspring and his brothers (p_{OMS}); a male offspring and another mother's sons (p_{OMM})

References

Bulmer, M.G. (1994). *Theoretical evolutionary ecology*. Sinauer Associates, Sundarland, MA.

Christiansen, F.B. (1991). On the conditions for evolutionary stability for a continuously varying character. *Am. Nat.*, 138, 37-50.

Eshel, I. (1996). On the changing concept of evolutionary population stability as a reflection of a changing point of view in the quantitative theory of evolution. *J. Math. Biol.*, 34, 485-510.

Frank, S.A. (1998). *Foundations of Social Evolution*. Princeton Univ. Press, Princeton, NJ.

Grafen, A. (2006). A theory of Fisher's reproductive value. *J. Math. Biol.*, 53, 15-60.

Otto, S.P. & Day, T. (2007). *A Biologist's Guide to Mathematical Modelling in Ecology and Evolution*. Princeton Univ. Press, Princeton, NJ.

Rodrigues, A.M.M. & Gardner, A. (2012). Evolution of helping and harming in heterogeneous populations. *Evolution*, 66, 2065-2079.

Rodrigues, A.M.M. & Gardner, A. (2013). Evolution of helping and harming in heterogeneous groups. *Evolution*, 67, 2284–2298.

Taylor, P.D. (1989). Evolutionary stability in one-parameter models under weak selection. *Theor. Pop. Biol.*, 36, 125-143.

Taylor, P.D. (1992). Altruism in viscous populations – an inclusive fitness model. - Evol. Ecol., 6, 352-356.

Taylor, P.D. & Frank, S.A. (1996). How to make a kin selection model. *J. Theor. Biol.*, 180, 27-37.
